# Analysis of several key factors influencing deep learning-based inter-residue contact prediction

**DOI:** 10.1093/bioinformatics/btz679

**Published:** 2019-08-30

**Authors:** Tianqi Wu, Jie Hou, Badri Adhikari, Jianlin Cheng

**Affiliations:** Department of Electrical Engineering and Computer Science, University of Missouri, Columbia, MO 65211, USA; Department of Electrical Engineering and Computer Science, University of Missouri, Columbia, MO 65211, USA; Department of Mathematics and Computer Science, University of Missouri, St. Louis, MO 63121, USA; Department of Electrical Engineering and Computer Science, University of Missouri, Columbia, MO 65211, USA

## Abstract

**Motivation:**

Deep learning has become the dominant technology for protein contact prediction. However, the factors that affect the performance of deep learning in contact prediction have not been systematically investigated.

**Results:**

We analyzed the results of our three deep learning-based contact prediction methods (MULTICOM-CLUSTER, MULTICOM-CONSTRUCT and MULTICOM-NOVEL) in the CASP13 experiment and identified several key factors [i.e. deep learning technique, multiple sequence alignment (MSA), distance distribution prediction and domain-based contact integration] that influenced the contact prediction accuracy. We compared our convolutional neural network (CNN)-based contact prediction methods with three coevolution-based methods on 75 CASP13 targets consisting of 108 domains. We demonstrated that the CNN-based multi-distance approach was able to leverage global coevolutionary coupling patterns comprised of multiple correlated contacts for more accurate contact prediction than the local coevolution-based methods, leading to a substantial increase of precision by 19.2 percentage points. We also tested different alignment methods and domain-based contact prediction with the deep learning contact predictors. The comparison of the three methods showed deeper sequence alignments and the integration of domain-based contact prediction with the full-length contact prediction improved the performance of contact prediction. Moreover, we demonstrated that the domain-based contact prediction based on a novel *ab initio* approach of parsing domains from MSAs alone without using known protein structures was a simple, fast approach to improve contact prediction. Finally, we showed that predicting the distribution of inter-residue distances in multiple distance intervals could capture more structural information and improve binary contact prediction.

**Availability and implementation:**

https://github.com/multicom-toolbox/DNCON2/.

**Supplementary information:**

Supplementary data are available at *Bioinformatics* online.

## 1 Introduction

Evolutionary variation in protein sequences is constrained by protein function and structure. Observed correlated mutation patterns in the sequences of a protein family indicate the direct physical contact between residue pairs in its 3D structure (Altschuh *et al.*, 1988), which can be used for inter-residue contact prediction (Gobel *et al.*, 1994). An approximate 3D protein structure can be built with good accuracy if a sufficient amount of accurately predicted residue–residue contacts are available (Marks *et al.*, 2011; Monastyrskyy *et al.*, 2014). Therefore, the contact distance-based *ab initio* 3D protein structure modeling calls for the development of more precise and sensitive contact prediction (Adhikari and Cheng, 2018; Brunger *et al.*, 1998; Hou *et al.*, 2019; Michel *et al.*, 2017; Pollastri and Baldi, 2002).

One of the key factors determining the quality of the correlated mutation information for contact prediction is the number of effective sequences (Neff) in a multiple sequence alignment (MSA). Due to the advancement in the DNA/RNA sequencing technology (Meyer *et al.*, 2008; Wilke *et al.*, 2016), a large number of sequences are available in public databases, making it possible for characterizing correlations between residue pairs of many proteins more accurately for contact prediction than before. However, correlated residue pairs within a protein are not necessarily close in its 3D structure. Some of them may reflect the functional constraints without structural implication and some of them may be accidental indirect correlated mutations due to transitive effects (Weigt *et al.*, 2009). In order to disentangle the directly correlated couplings from the indirect ones, several direct-coupling analysis methods were developed, such as plmDCA (Ekeberg *et al.*, 2013), GREMLIN (Kamisetty *et al.*, 2013) and CCMpred (Seemayer *et al.*, 2014), and the sparse inverse covariance estimation method PSICOV (Jones *et al.*, 2012). The residue–residue coevolutionary scores generated by these methods were also used as features with neural networks to predict contacts (Jones *et al.*, 2015).

Apart from the direct correlated mutation (or coevolutionary) analysis, deep learning is a major technological innovation to improve contact prediction. After deep learning contact prediction was first introduced in 2012 (Di Lena *et al.*, 2012; Eickholt and Cheng, 2012), different deep learning architectures have been designed to integrate traditional sequence features with residue–residue coevolutionary scores to substantially improve the accuracy of contact prediction (Adhikari *et al.*, 2018; Hanson *et al.*, 2018; Jones and Kandathil, 2018; Skwark *et al.*, 2014; Wang *et al.*, 2017). However, except for deep learning architecture itself, other factors that affect the performance of deep learning-based contact prediction methods directly or indirectly have not been systematically studied.

In this work, we analyzed the results of our three deep learning-based contact prediction methods in the CASP13 experiment and explained why they significantly outperformed the coevolution-based methods (e.g. CCMpred). We demonstrated how the contact distance distribution prediction helped improve the performance of contact prediction and investigated how the Neff in MSAs, MSA generation protocols and domain parsing method contributed to the contact prediction improvement.

## 2 Materials and methods

### 2.1 Deep network architecture for protein inter-residue contact prediction

Our MULTICOM contact predictors were based on the same deep learning architecture of DNCON2 trained on the same dataset (see details in Adhikari *et al.*, 2018) but used different approaches to generate input and process output during prediction, which was a good setting for studying the external factors influencing the accuracy of deep learning-based contact prediction. The common deep learning architecture consists of six convolutional neural networks (CNNs), which are used at the two stages. Each CNN network model contains 6 hidden convolutional layers with 16 5 × 5 filters and an output layer with one 5 × 5 filter to predict a contact probability map at any distance threshold. At the first stage, the 27 features including scalar features (e.g. protein length), 1D features (e.g. secondary structure prediction) and 2D features [e.g. residue–residue coevolutionary scores calculated by coevolution-based methods CCMpred, FreeContact (in EVfold-mfDCA mode) (Kajan *et al.*, 2014) and PSICOV] are transformed into 56 2D features (see details in Adhikari *et al.*, 2018). Using these 56 2D features as input, five of the six CNN models are used to predict contact maps at five distance thresholds: 6 Å, 7.5 Å, 8 Å, 8.5 Å and 10 Å, resulting in the predicted distance distribution of residue pairs. At the second stage, the sixth CNN model takes the predicted distance distributions and the original features as input to make final short-range, medium-range and long-range contact predictions in one contact map at the distance threshold of 8.0 Å.

### 2.2 MSA generation, template-based domain parsing, full-length and domain-based contact prediction

The coevolutionary feature calculated from MSA is one of the most important features for the contact prediction. In CASP13, we applied different alignment combination strategies to the three MULTICOM server predictors. MULTICOM-NOVEL used Jackhmmer (Johnson *et al.*, 2010) to search sequences against Uniref90 with four E-value thresholds (from E^−20^, E^−10^, E^−4^ to 1). If some alignment contains ≥5L sequences (L: target sequence length), the one at the lowest E-value threshold was used; otherwise, the alignment at E-value of 1 was used. MULTICOM-CONSTRUCT first ran HHblits (Remmert *et al.*, 2011) with the coverage threshold of 60% to search against Uniclust30. If the number of sequences is less than 2000, it further ran Jackhmmer on Uniref90 to collect more sequences. After comparing the alignment from HHblits search, Jackhmmer search and their combination, it selected the alignment with the highest Neff to generate coevolution features. MULTICOM-CLUSTER applied a similar strategy as MULTICOM-CONSTRUCT, except that it added one extra step of filtering out redundant sequences at 90% sequence identity threshold using the MMseqs2 linear time clustering algorithm (Steinegger and Soding, 2017).

MULTICOM-CONSTRUCT and MULTICOM-CLUSTER also predicted domain boundaries by searching a target against the non-redundant protein template sequences from the Protein Data Bank. The region mapped to homologous templates was considered as template-based modeling (TBM) domain, otherwise template-free modeling (FM) domain. For each FM domain, the two predictors searched the domain sequence against the databases to generate MSA for the domain as they did for the full-length target sequence. The full-length MSA was used to predict full-length contact maps and the domain-based MSA was used for predicting the domain-based contact maps. The predicted contact probabilities in the full-length contact map were replaced by their counterparts in the domain-based contact map if the latter were larger than the former. We also implemented the same domain splitting approach on DNCON3 that applied PSIBLAST and Jackhmmer to generate alignments. Our human contact predictor MULTICOM averaged contact predictions from our four server predictors. However, because DNCON3 and MULTICOM missed some CASP13 targets, they were not discussed in this work.

### 2.3 *Ab initio* domain parsing based on the plot of the number of sequences in MSA

The template-based domain parsing is time consuming and complicated. During the CASP13 experiment, we tested a simple method to directly detect domains or more strictly regions that have a significantly lower number of sequences than other regions in an MSA, aiming to search the individual domain/region against the sequence database again to collect more homologous sequences in order to generate better coevolutionary features for them. Specifically, we computed the number of sequences covering each residue position of a target sequence in an MSA. Using the median of the numbers as a cutoff, we defined a consecutive sequence fragment (with the length at least 30 residues) in the full-length sequence as a hard domain if the number of sequences of all the residue positions in the fragment was less than the cutoff value. The contact prediction of a domain was integrated with the full-length contact predictions by MULTICOM-CLUSTER and MULTICOM-CONSTRUCT as in Section 2.2.

### 2.4 Dataset and evaluation metrics

We evaluated our contact predictors on all these 108 structural domains of 75 official CASP13 targets, including 43 FM and FM/TBM domains. To be consistent with the CASP convention, contact predictions were evaluated at domain-level. We used ConEVA (Adhikari *et al.*, 2016) to analyze contact prediction results and also referred to the assessments published at the CASP website (https://predictioncenter.org/). The emphasis of the evaluation was placed on the precision of long-range contacts with sequence separation ≥24 residues.

We used the Neff (e.g. non-redundant sequences), Neff, to estimate the quality of alignments. It was calculated as the following function:

(1) $$N_{\text{eff}}=\sum_{i=1}^{N}\frac{1}{{\text{weight}}_{i}}$$

where *N* was the number of sequences in an MSA. Assuming ${\mathrm{weight}}_{\mathrm{ij}}$ was the sequence identity between any two homologous sequences *i* and *j* in the MSA. It was equal to the maximum value 1 if and only if the sequence identity between *i* and *j* is ≥62%. For each sequence *i* in the MSA, ${\mathrm{weight}}_{i}$ was the sum of ${\mathrm{weight}}_{\mathrm{ij}}$ over all the sequences (including itself) in the MSA. Neff was the sum of $\frac{1}{{\;\mathrm{weight}}_{i}}\;$over all the sequences in the MSA. Higher ${\mathrm{weight}}_{i}$, smaller the Neff. To calculate the Neff at the domain level, we first extracted the sequence alignment of a domain from a full-length sequence alignment of the whole target and then calculated the Neff of the domain according to Equation (1).

## 3 Results

### 3.1 Impact of deep learning on improving coevolutionary input for contact prediction

We follow the CASP definition that a pair of residues are in contact if the distance between their C_β_ atoms in the native structure is below 8.0 Å. A contact is considered long-range if the sequence separation between the two residues of the contact is at least 24 residues away in the protein sequence. A contact with sequence separation between 12 and 23 residues is at medium range and between 6 and 11 residues at short range. We focus on evaluating the precision of top L/5, L/2 and L predicted long-range contacts ranked by contact probability scores. L is sequence length. We compare our deep learning predictors with three coevolution methods, including CCMpred (based on pseudo-likelihood maximization), Freecontact (based on direct coupling analysis) and PSICOV (sparse inverse covariance estimation). The results are shown in Table 1. The input for those three coevolution methods is the sequence alignment generated by MULTICOM-NOVEL alignment pipeline.

**Table 1.** Contact prediction precision on 108 CASP13 domains

Method	Short-range (%)			Medium-range (%)			Long-range (%)		
	Top-L/5	Top-L/2	Top-L	Top-L/5	Top-L/2	Top-L	Top-L/5	Top-L/2	Top-L
MULTICOM-CLUSTER	65.8	45.3	28.5	69.0	52.7	35.9	70.7	58.3	45.3
MULTICOM-CONSTRUCT	66.2	45.1	28.2	67.6	51.5	35.1	68.2	57.3	44.3
MULTICOM-NOVEL	61.6	42.8	27.1	64.1	47.8	32.7	62.6	52.2	41.0
CCMpred	31.1	20.7	14.7	38.3	25.0	17.5	43.4	33.9	24.9
Freecontact	25.7	17.9	12.9	32.0	22.6	15.6	36.2	27.0	20.1
PSICOV	27.8	18.8	14.2	32.3	21.5	16.0	38.1	29.2	21.3

**Fig. 1.**
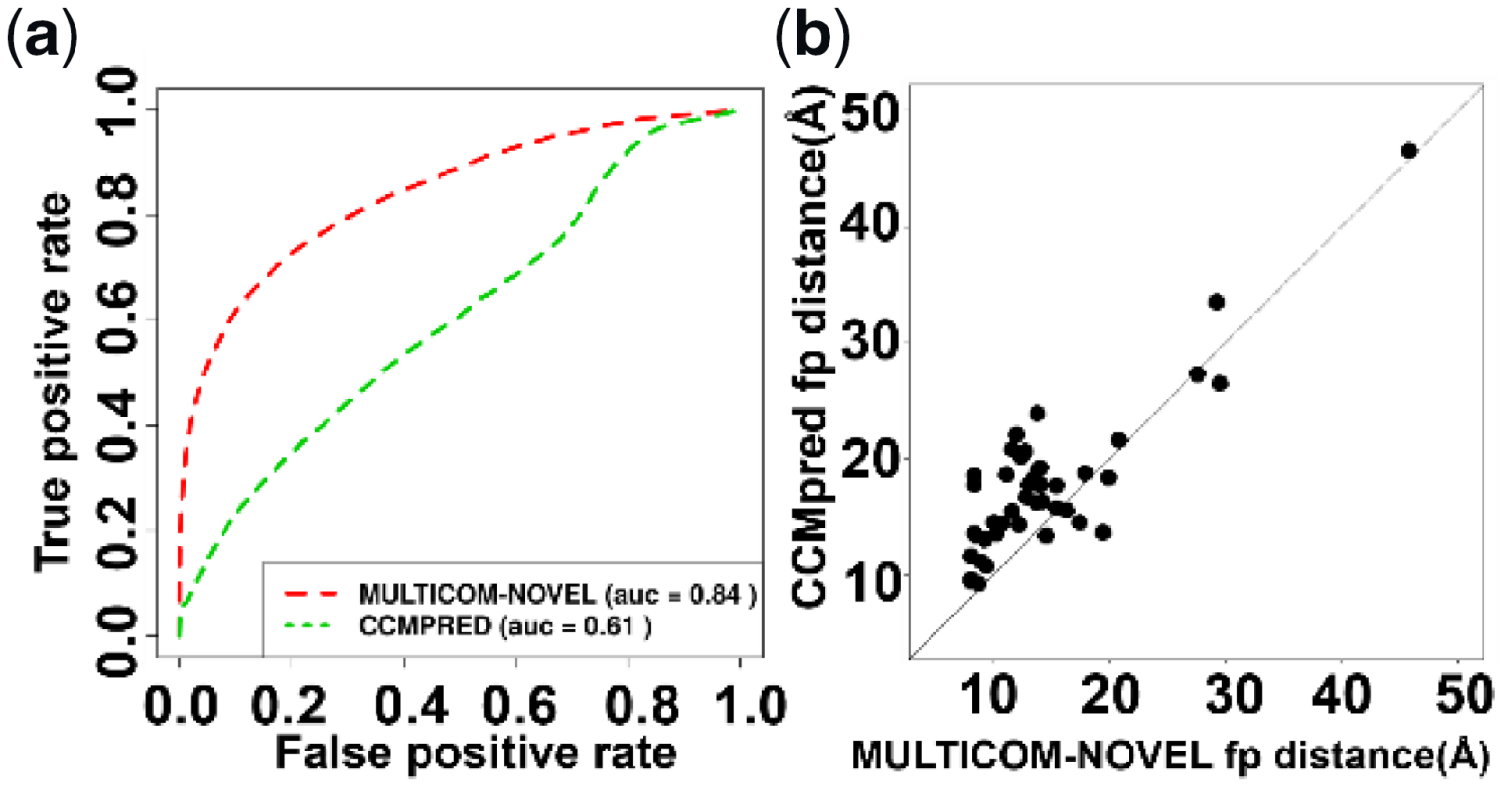
Contact prediction performance of MULTICOM-NOVEL and CCMpred. (**a**) ROC curve of CCMpred on the long-range predicted contacts of 43 CASP13 FM and FM/TBM targets are shown in green and MULTICOM-NOVEL in red. Deep learning-based method, MULTICOM-NOVEL, greatly improves the AUC score from 0.61 to 0.84. (**b**) The plot of the average distance of false positive contact predictions made by MULTICOM-NOVEL versus CCMpred for each CASP13 FM and FM/TBM target (denoted by a dot in the plot). The average distance of false positive contacts over all the targets for MULTICOM-NOVEL is 14.1 Å, smaller than that for CCMpred (17.8 Å)

It shows that the deep learning-based methods outperformed the coevolution-based methods on all ranges (short, medium or long) for top L/5, top L/2 and top L predicted contacts. For instance, for top L/5 long-range predicted contacts, the precision of the best performing predictor—MULTICOM-CLUSTER was 70.7%, substantially higher than 43.4% of CCMpred, 36.2% of Freecontact and 38.1% of PSICOV. Figure 1a illustrates the ROC curves of MULTICOM-NOVEL (a full-length deep learning contact predictor without domain combination) and CCMpred on 43 CASP13 FM and FM/TBM targets. The AUC score (the area under the ROC curve) of MULTICOM-NOVEL was 0.84, much higher than 0.61 of CCMpred. Figure 1b plots the average real distances of false positive (fp) contact predictions of CCMpred against those of MULTICOM-NOVEL for each of 43 FM and FM/TBM targets, indicating the fps of CCMpred had larger average distances than those of MULTICOM-NOVEL for most targets.

**Fig. 2.**
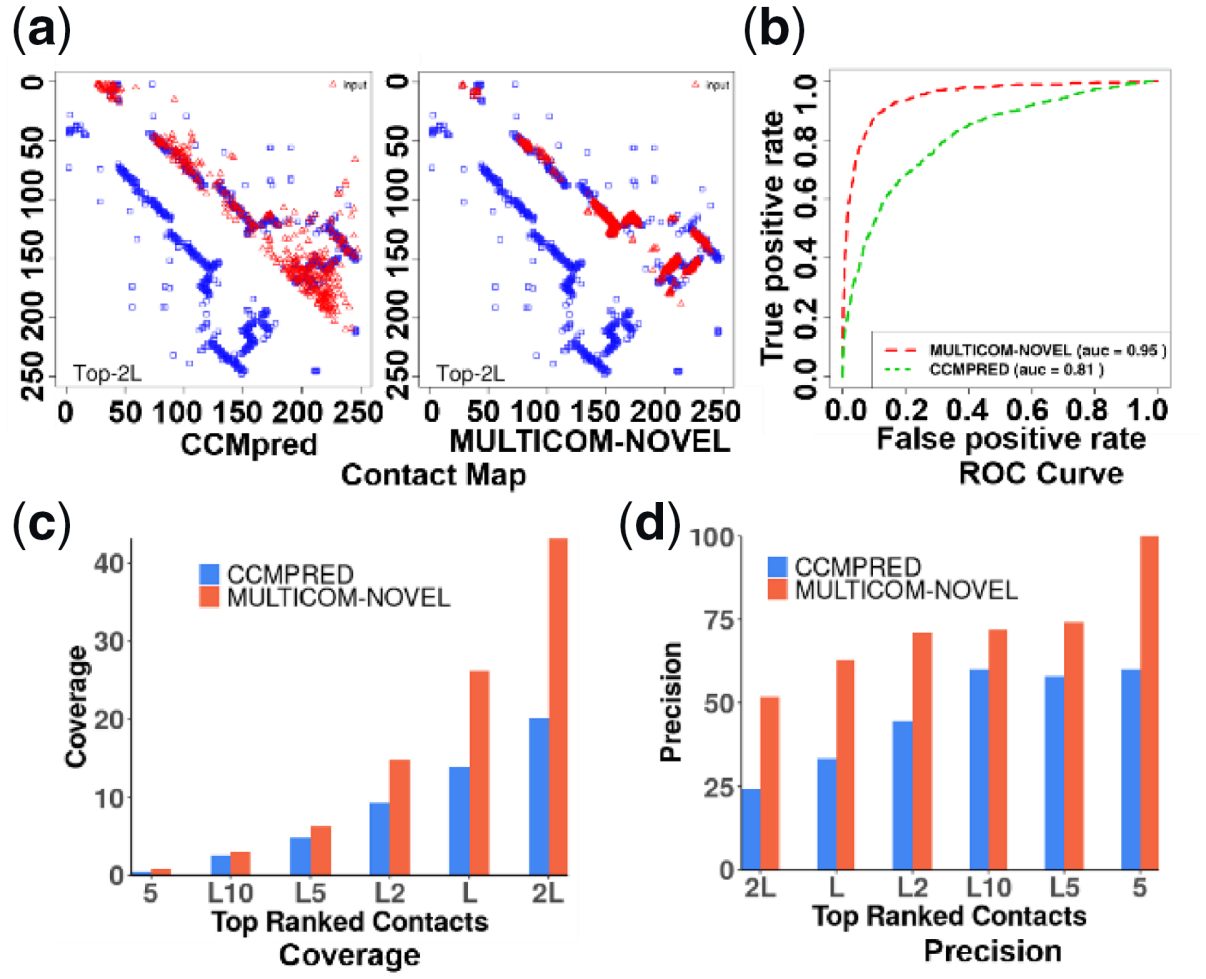
Contact prediction results of CCMpred and MULTICOM-NOVEL for target T0953s2. (**a**) Top 2L long-range contacts predicted by the two methods (red) versus true contacts (blue); (**b**) ROC curves of the two methods (red: MULTICOM-NOVEL, AUC = 0.95; green: CCMpred, AUC = 0.81); (**c**) The coverage (i.e. 100 * TP/N, where, TP is number of true positive contacts and *N* is number of native contacts) of top 5 to top 2L long-range contacts predicted by the two methods. (**d**) The plot of precision of predicted top 5, top L/10, top L/5, top L/2, top L and top 2L long-range contacts of the two methods. (Color version of this figure is available at *Bioinformatics* online.)

One reason that the deep learning predictors perform much better is that the CNN is able to detect the correlation between contacts within a certain region in the input matrices (e.g. a group of contacts between two beta strands). The size of the region depends on the size of the total receptive field captured by convolutions (Jones and Kandathil, 2018). For instance, the total receptive field of one CNN with seven convolutional layers and 5 × 5 filters in MULTICOM-NOVEL is 29 × 29 (i.e. 5 + 4 * 6). The whole deep network of the two stages has the total receptive field with size of 53 × 53 (i.e. 5 + 4 * 12). The more convolution layers we have, the larger the receptive field sizes are, and the more global the method is. Therefore, the deep learning predictors are global machine learning methods like early 2D recurrent neural network methods for contact prediction (Pollastri and Baldi, 2002; Tegge *et al.*, 2009). In contrast, local coevolution methods only use the coevolutionary scores of each pair of residues to predict contact, ignoring other correlated contacts. Leveraging the correlation between contacts by the global deep learning methods can filter out dangling false contacts without forming clusters with other contacts or recall some missing contacts in a cluster of contacts. Figure 2a compares a contact map predicted by CCMpred and that predicted by MULTICOM-NOVEL illustrating such an effect. A lot of noises (fps) in the contact map of CCMpred were removed in the contact map of MULTICOM-NOVEL, leading to a cleaner map. And some missing contacts in the contact map of CCMpred were recalled in the contact map of MULTICOM-NOVEL, leading to denser contact clusters. Figure 2(b–d) shows that the contact predictions of this target made by MULTICOM-NOVEL have higher AUC score, coverage and precision than CCMpred.

### 3.2 Impact of Neff and alignment quality on precision

**Fig. 3.**
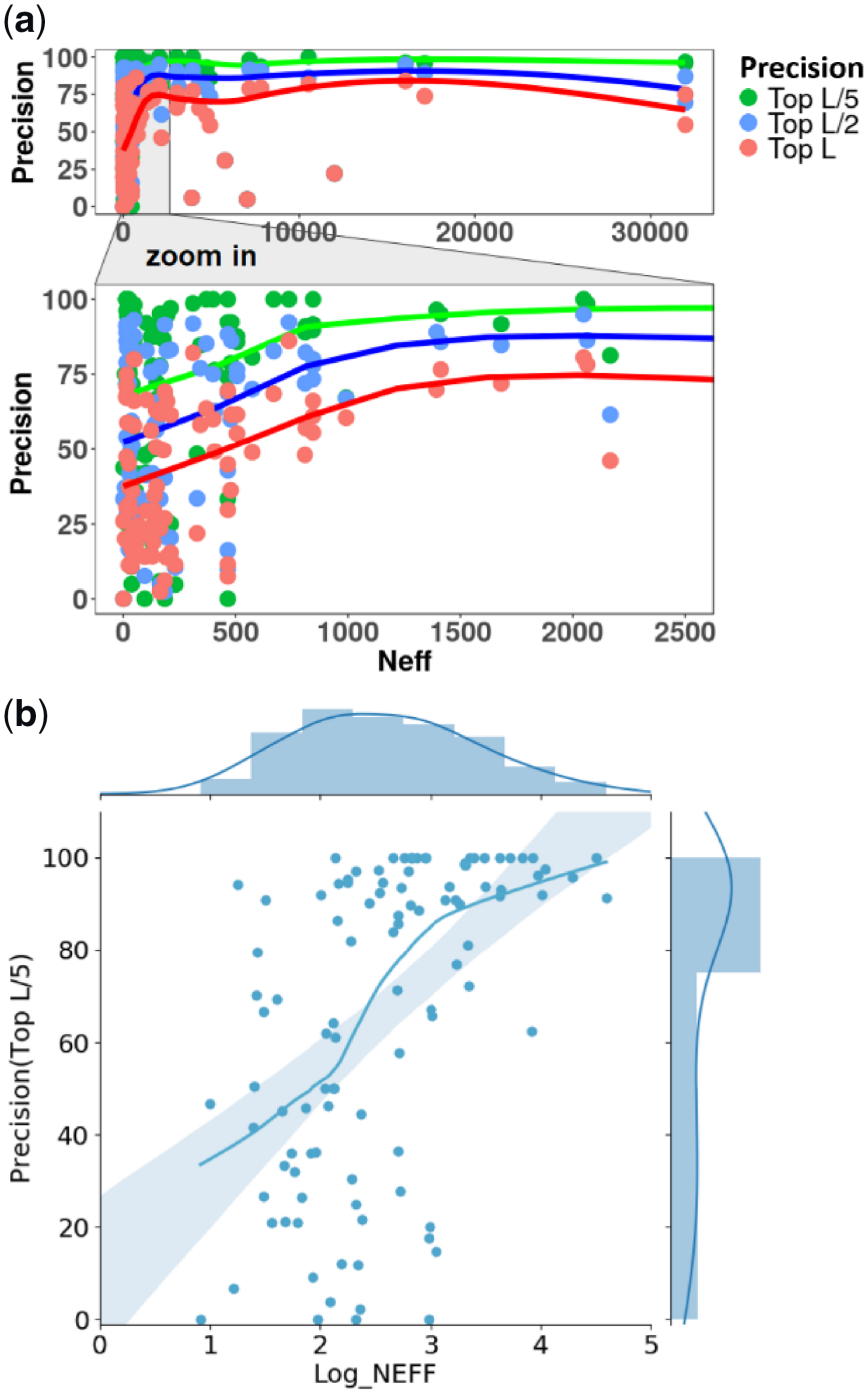
(**a**) Plot of contact prediction precision against Neff of multiple sequence alignments for 108 CASP13 domains for MULTICOM-NOVEL. Dots with different colors represent precisions of different numbers of long-range contact predictions (top L/5, top L/2 and top L). The curve is the LOESS line fitting the dots. The plot in Neff range [1, 2500] is zoomed in. (**b**) Scatterplot of the precision of top L long-range contact predictions versus log (Neff) with the marginal histograms of the precision and log (Neff) shown on the top and on the right, respectively. The curve is the LOESS line fitting the dots. (Color version of this figure is available at *Bioinformatics* online.)

The coevolution-based feature is the most important input feature for contact prediction. The reliable calculation of coevolutionary scores requires a large number of diverse homologous sequences in an MSA. We found that deeper alignment with more diverse homologous sequences (higher Neff) usually resulted in higher contact prediction precision for the deep learning predictors, which is expected because higher Neff generally leads to better coevolutionary input features for them. Figure 3a is the plot of the precisions of top long-range contact predictions made by MULTICOM-NOVEL against Neff on 108 CASP13 domains (see the plot at logarithm scale in Supplementary Figure S1). Based on the results from MULTICOM-NOVEL, the minimum number of sequences in the MSA that led to a top L/5 long-range contact prediction precision larger than 50% was 13 and the minimum Neff is only 8. The fitting curve in Figure 3a shows that the precision increases as Neff increases until it largely saturates when Neff is higher than 550. The Pearson's coefficient between the logarithm of Neff and the precision of top L/5 long-range contact precisions is 0.53 and *P*-value is 6.5E-8 under the hypothesis that the correlation coefficient is 0, indicating the correlation between Neff and contact precision is significant.

Neff of MSAs depends on the alignment methods used with the deep learning predictors. To compare the quality of MSAs generated by the three predictors, we ran CCMpred, Freecontact and PSICOV on their alignments to generate coevolution-based contact predictions and evaluated the precision of top L/5 long-range predicted contacts. Supplementary Table S1 shows the contact prediction results of the three methods on 43 CASP13 FM and FM/TBM targets. For all three predictors (CCMpred, Freecontact and PSICOV), the average precision of MULTICOM-CLUSTER alignment was higher than MULTICOM-CONSTRUCT and MULTICOM-NOVEL. The better quality of MULTICOM-CLUSTER alignment than that of MULTICOM-CONSTRUCT indicated the filtering out redundant sequences in MSAs improved the alignment quality and therefore the direct coevolutionary input for the deep learning contact predictor. The difference in the input could largely explain why the final precision (70.7%) of MULTICOM-CLUSTER was higher than that (68.2%) of MULTICOM-CONSTRUCT and that (62.6%) of MULTICOM-NOVEL for top L/5 long-range contact predictions (Table 1).

### 3.3 Impact of domain-based contact prediction

It has been shown that integrating domain-level contact predictions with full-length contacts can improve contact prediction precision, especially when domain boundaries can be accurately identified (Buchan and Jones, 2018). The reason is that the full-length sequence search may be dominated by the domain that has a lot of similar homologous sequences, causing other domains, particularly hard domains to have much fewer sequences to be found. Therefore, it is necessary to search the sequence of under-represented domains against the sequence database to collect more sequences for them.

We used the two domain parsing methods (a template-based method and an *ab initio* method) to split the full-length sequence of a target into domains. We evaluated the performance of two domain-parsing methods with MULTICOM-CLUSTER along with its full-length contact prediction on the domains of all 65 CASP13 FM, FM/TBM and TBM-hard domains (Table 2). The precision of top L/5 long-range predicted contacts of using the template-based domain parsing, the *ab initio* domain parsing and the full-length alignments without domain parsing was 58.4%, 54.9% and 52.5%. Using domain parsing clearly improved the contact prediction by improving the coevolution input features of some domains. The detailed changes of precision on those targets are shown in the Supplementary Table S6. On average, most improvement caused by the domain parsing occurred on TBM-hard targets rather than on FM and FM/TBM targets. The template-based domain parsing helped substantially improve the precision on seven domains [T0989-D2 (FM), T0964-D1 (TBM-hard), T0981-D1 (TBM-hard), T0981-D4 (TBM-hard), T0981-D4 (TBM-hard), T0981-D5 (TBM-hard) and T0999-D2 (TBM-hard)]. The precision of the *ab initio* domain parsing was 2.4 percentage point on average better than not using domain parsing for top L/5 contact predictions but was worse than the template-based domain parsing probably because its boundary prediction was less accurate (Table 2). However, unlike the template-based domain parsing that required known structural templates as input and was much more complicated, the *ab initio* domain parsing was purely sequence-based and much easier to implement.

**Table 2.** Precisions of top L/5, L/2, L long-range contacts of the two domain parsing methods and full-length method in MULTICOM-CLUSTER on 65 CASP13 domains

Precision	Template-based domain parsing (%)	*Ab initio* domain parsing (%)	Full length (%)
Top L/5	58.4	54.9	52.5
Top L/2	46.4	44.3	43.1
Top L	35.9	34.1	33.4

**Fig. 4.**
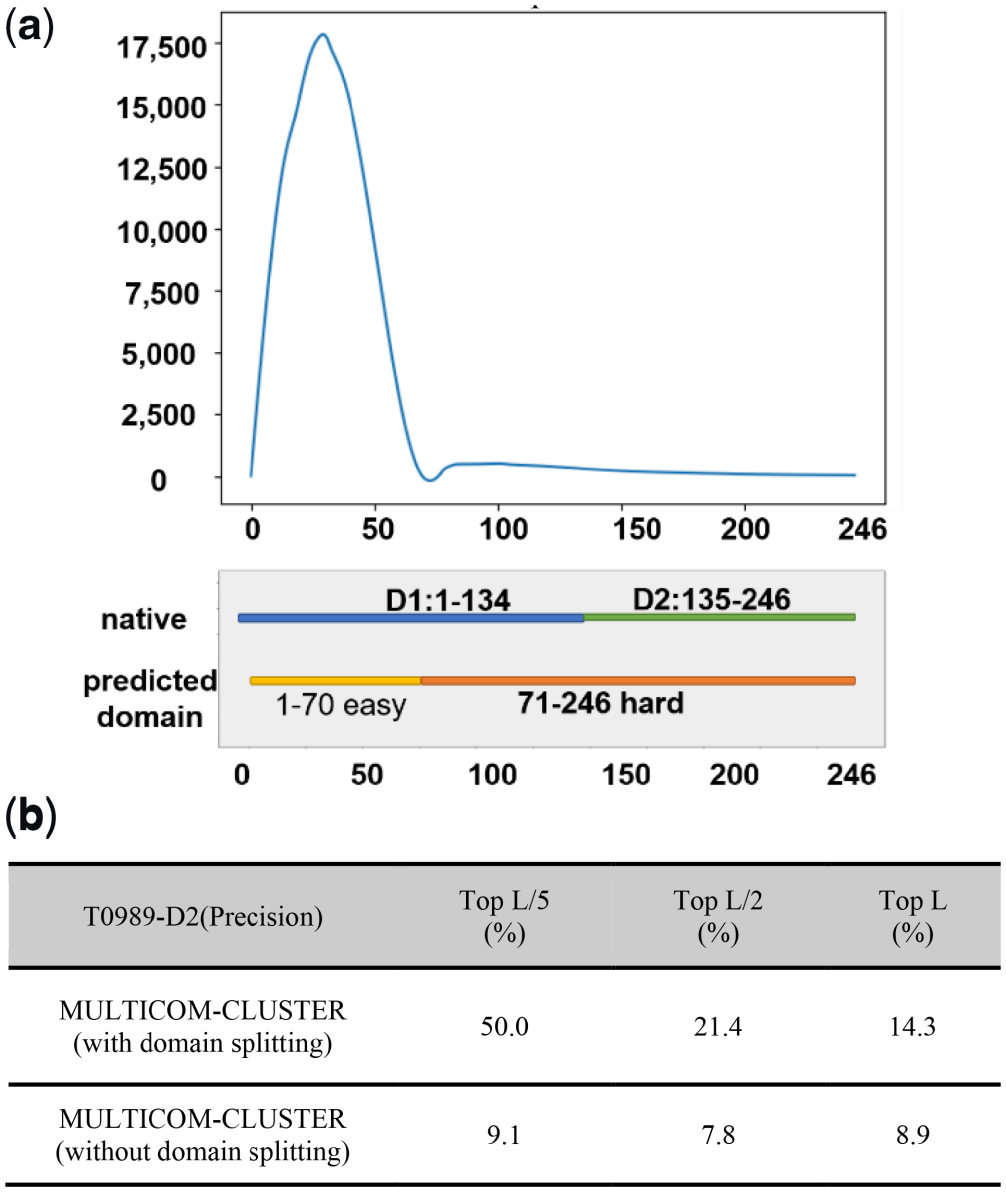
Domain parsing and domain-based contact prediction of target T0989. (**a**) Plot of number of sequences in the MSA of T0989 against residue positions, true domain boundaries and the boundaries predicted by the *ab initio* domain parsing method. (**b**) The contact prediction precision for the second domain of T0989 by MULTICOM-CLUSTER with/without the domain parsing and integration of domain-based contact prediction

The only input needed for *ab initio* domain parsing was the MSA. It detected potential domain boundaries directly based on the distribution of number of sequences at each residue position in the MSA. Figure 4 is one example (target T0989) to demonstrate how the *ab initio* domain parsing used the positional information obtained from the MSA to identify potential domain boundaries. T0989 is split into two real domains. Its first domain goes from Residue 1 to Residue 134. The second domain goes from Residue 135 to Residue 246. We calculated the number of sequences at each residue position in the MSA and plotted the numbers against their positions (Fig. 4a). This target was split into two sequential domains (1–70 and 70–246). The full-length alignment had a low number of sequences for the second predicted domain (70–246), which covered the range of the second domain in the true structure. For comparison, we compared the contact prediction results for domain T0989-D2 (135–246 in true structure), with and without domain parsing and integration of domain-based contact prediction (Fig. 4b). The precision with the domain-based contact prediction for top L/5 predicted contacts was 50.0%, much higher than 9.1% without domain-based contact prediction.

### 3.4 Impact of contact distance distribution prediction

The two-stage CNN architecture used in our deep learning methods predicted the distribution of inter-residue distance in the range (0 Å, 6 Å, 7.5 Å, 8 Å, 8.5 Å, 10 Å, +∞) at the first stage, which was used as input together with the original input features to predict the final contact map at the second stage. On the 108 domains of 75 CASP13 targets, we compared the contact prediction precision of using or not using the predicted contact distance distribution as the extra input (Table 3).

**Table 3.** Top L/5 long-range contact precision of two stages by MULTICOM-CLUSTER full-length prediction for 108 CASP13 domains

Precision	Stage 1	Stage 1	Stage 1	Stage 1	Stage 1	Stage 2
	6.0 Å (%)	7.5 Å (%)	8.0 Å (%)	8.5 Å (%)	10.0 Å (%)	8.0 Å (%)
Top L/5	52.4	56.7	58.3	56.9	54.4	63.7
Top L/2	43.3	47.6	47.2	47.6	45.7	52.4
Top L	33.5	36.7	36.1	36.7	35.5	40.6

The result shown in Table 3 exhibits a significant increase (from 58.3% to 63.7%) in precision of top L/5 predicted contacts at 8 Å threshold by applying two-level CNN model methods in comparison. The results demonstrated that predicting the distribution of inter-residue distance provided the valuable information to improve the precision of contact prediction.

**Fig. 5.**
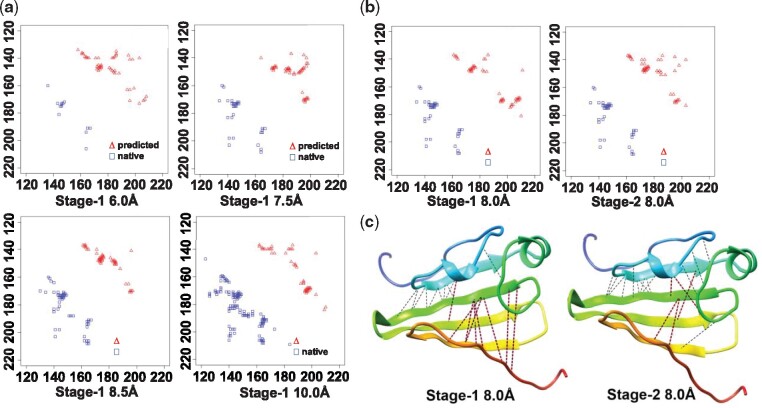
Top L/2 long-range predicted contacts for T0963-D3 at Stage 1 without the inter-residue distance distribution as input and at Stage 2 with the inter-residue distance distribution as input. (**a**) Top L/2 long-range predicted contacts (red) versus true contracts (blue) for T0963-D3 at Stage 1 at distance thresholds of 6, 7.5, 8.5 and 10 Å. (**b**) Top L/2 long-range contacts versus true contacts at the distance threshold of 8.0 Å at Stage 1 and Stage 2. (**c**) The predicted top L/5 long-range contacts at the distance threshold of 8.0 Å at Stage 1 and Stage 2 are visualized on the native structure of target T0963-D3. The red lines in the structure are the false positive contacts and the black lines are true positive contacts. (Color version of this figure is available at *Bioinformatics* online.)

Figure 5 illustrates the impact of the predicted distance distribution on the final contacts prediction of the domain T0963-D3. Figure 5a shows the predicted contact maps at several different thresholds at Stage 1 without using the distance distribution as input, which capture slightly different structural patterns. Figure 5b is a comparison between the contact map at 8 Å of Stage 1 and that of Stage 2. Figure 5c shows a side-by-side comparison of the top L/5 predicted long-range contacts visualized in the true tertiary structure between Stage 1 and Stage 2 at the distance threshold of 8 Å. The fp contacts, whose distance is greater than 8 Å in true tertiary structure, are labeled as red line segments, while the black ones denote correctly predicted contacts. By adding the predicted contact distance distribution as input at Stage 2, more true positive contacts were recalled in the true structure as shown in Figure 6c. The final precision was increased from 36.8% to 68.4% for top L/5 predicted long-range contacts and from 23.4% to 42.6% for top L/2 predicted long-range contacts. This example confirms that it is useful to predict the detailed inter-residue distance distribution in multiple distance intervals, which is more informative than using binary contact maps at one threshold (e.g. 8 Å) as previously demonstrated in (Adhikari *et al.*, 2018).

### 3.5 Impact of protein sequence database on contact prediction—a post-CASP13 analysis

The size of protein sequence databases affects how many homologous sequences may be found for a target. After the CASP13 experiment, we noticed that some groups used the metagenomics sequence database not used with our predictors to find more homologous sequences for some CASP13 targets than our methods. After CASP13, we updated the alignment generation pipeline MULTICOM-CLUSTER to include an extra search on a large metagenomic protein sequence database Metaclust50 that was built from 1.6 billion metagenomic sequence fragments with ≤50% sequence identity (Steinegger and Soding, 2018). MULTICOM-CLUSTER first searched HHblits against Uniclust30 sequence database, and then ran Jackhmmer on Uniref90 sequence database to collect more homologous sequences. The alignment generated by HHblits and Jackhmmer was used to build an HMM model profile. Then, it applied HMMER-3.1b2 hmmsearch program to search the profile against Metaclust50 for more homologous sequences. Finally, it combined the MSAs from HHblits/Jackhmmer searches and hmmsearch search and removed duplicates in them. The combined MSA was used to predict contacts. We compare the contact prediction performance of MULTICOM-CLUSTER with the alignment generation pipeline before and after adding an extra dataset, Metaclust50. The precisions shown in Table 4 are the average top L/5, top L/2 and top L long-range contact precision of 43 CASP13 FM and FM/TBM domains, which indicates that enlarging the sequence database is helpful for increasing contact prediction precision as initially demonstrated in (Ovchinnikov *et al.*, 2017).

**Table 4.** Comparison of contact prediction precision by MULTICOM-CLUSTER before and after adding Metaclust50 on CASP13 43 FM and FM/TBM domains

Precision	Top-L/5 (%)	Top-L/2 (%)	Top-L (%)
MULTICOM-CLUSTER (adding Metaclust50)	55.0	42.5	33.1
MULTICOM-CLUSTER	51.0	40.9	31.6

### 3.6 When does deep learning work or not work?

The deep learning predictors generally tend to work well if there is a sufficient number of diversely related homologous sequences available. However, the sufficiency is target-specific. When there are fewer homologous sequences available for a target, they can still leverage weak signals in shallow alignments and perform well in some cases. Table 5 shows the performance of MULTICOM-NOVEL on CASP13 domains with shallow alignments (e.g. the number of sequences in the alignment < 200 or Neff < 100). On 7 out of 13 targets, the precision for top L/5 contact predictions was ≥75%, while it did not perform well on the remaining 6 cases. If no diverse homologous sequence can be found at all (Neff = 1), alignment-related features as coevolutionary scores are certainly not reliable. The deep learning methods largely rely on other features as secondary structure and solvent accessibility to make prediction. For instance, two targets T0991 and T1008 only had one sequence in their alignment. Therefore, their alignment-related features as MSA statistics (Shannon entropy sum, mean and mutual information) and coevolution-based features did not contain useful information. In those two extreme cases, the accuracy of secondary structure prediction may play an important role on contact prediction. For T0991-D1, the predicted secondary structure accuracy was only 41% and the accuracy of top L/5 long-range contact prediction was 0. In contrast, T1008-D1 had the secondary structure prediction accuracy of 75% and the top L/5 long-range contact precision was 43.75%. However, in our post-CASP13 experiment, by using the metagenomics database, the Neff of the MSA for T0991 was increased from 1 to 3. This small change improved the precision of ResPRE (Li *et al.*, 2019) that uses only alignments as input from 0% to 36.6%, suggesting adding just a few diverse homologous sequences can drastically improve the prediction accuracy.

**Table 5.** Precision of MULTICOM-NOVEL on CASP13 targets with shallow alignments (*N* ≤ 200 or Neff < 100)

Targets	*N*	Neff	Top L/5 Prec. (%)	Top L/2 Prec. (%)
T0957s2-D1	80	45	48.39	29.49
T0958-D1	46	17	25	28.21
T0987-D1	200	26	75.68	48.39
T0987-D2	200	27	75	41.84
T0990-D1	67	30	25	15.79
T0990-D3	67	36	30.23	17.76
T0991-D1	1	1	0	0
T0999-D2	121	25	94.51	87.67
T0999-D3	121	81	86.11	80
T0999-D4	121	67	97.96	82.79
T0999-D5	121	91	98.28	93.06
T1001-D1	13	8	75	37.14
T1008-D1	1	1	43.75	33.33

Moreover, a larger Neff in the alignment does not always lead to the increase of prediction accuracy with our deep learning predictors. For example, MULTICOM-NOVEL overperformed MUTICOM-CLUSTER by 22 percentage points (94.44% versus 72.22%) on T1015s1-D1 according to the precision of top L/5 contact predictions (Table 6), while the Neff from the former (146) was less than the latter (165).

**Table 6.** Precision of top L/5 long-range contact prediction of T1015s1-D1with different contact predictors

T1015s1-D1	Neff	Top-L/5 (%)
MULTICOM-CLUSTER	165	72.22
MULTICOM-NOVEL	146	94.44
CCMpred (MULTICOM-CLUSTER alignments)	165	61.11
CCMpred (MULTICOM-NOVEL alignments)	146	55.56
ResPRE (MULTICOM-CLUSTER alignments)	165	94.44
ResPRE (MULTICOM-NOVEL alignments)	146	94.44

We further applied two external methods, CCMpred and ResPRE (Li *et al.*, 2019) with the alignments from MULTICOM-CLUSTER and MULTICOM-NOVEL as the only input to make contact predictions for T1015s1. For both contact predictors, the precision of long-range top L/5 contact predictions from the alignments of MULTICOM-CLUSTER was higher than or equal to MULTICOM-NOVEL (Table 6), which indicated that alignment from MUTICOM-CLUSTER might contain more coevolutionary signals than MULITCOM-NOVEL. But the deep learning network used by MULTICOM-CLUSTER cannot leverage the information well, suggesting there is still a significant room of improving the training and design of deep learning models.

## 4 Conclusion and future work

We evaluated the performance of our three CASP13 deep learning-based contact prediction methods (MULTICOM-CLUSTER, MULTICOM-CONSTRUCT and MULTICOM-NOVEL) and investigated the key factors that affected their performance. We showed that leveraging residue–residue interactions and contact correlations within an area (i.e. the total receptive field) in the input matrices captured by multi-layers of convolutions was a key reason that the deep learning methods substantially outperformed the local contact prediction methods that treated each pair of residues independently. Through multiple layers of convolutional feature extraction, the deep learning methods could capture the long-range interactions between residues and correlations between contacts to improve contact prediction. Our experiment also demonstrated that predicting the distance distribution of inter-residue distance in multiple distance intervals was able to capture more structural information than a binary contact map at a single distance threshold. Leveraging the distance distribution can improve contact prediction.

The accuracy of contact prediction also depends on the number of effective (true) homologous sequences. Therefore, the sensitive sequence search protocol is important for further improving contact prediction. The Neff is also related to the size of protein sequence database. We confirmed that the metagenomics sequence database contained some sequences not in widely used Uniref90 and Uniclust30 databases and should be used if necessary.

Domain parsing is also important to improve contact prediction, which is especially useful for domains for which few homologous sequences are found during the full-length sequence search. We demonstrated that a fast, simple domain-based contact prediction methods based on MSAs alone could consistently improve contact prediction accuracy over the full-length contact prediction, even though it was less accurate than the domain-based contact prediction based on the template-based domain parsing.

It is worth noting that another key factor that affects the accuracy of inter-residue contact prediction is the architecture of deep learning models and how they are trained, which are not investigated in this work. In the CASP13 experiment, some external deep learning methods performed better than our predictors, partially due to the difference in the architecture design and training strategy (Kandathil *et al.*, 2019; Li *et al.*, 2019; Xu and Wang, 2019). The selection of training and test data may have a significant impact on contact prediction accuracy. Currently, the redundancy of training and test data is controlled by sequence identity rather than structural similarity. The protein structural classification information can be useful in preparing training and test datasets. Besides, the metric used to select our best deep learning model is based on the precision of top L/5 long-range contact predictions, which may not be the best metric for evaluating the whole contact map. In some cases, the top L/5 predicted contacts can be concentrated in a certain region. They may have high precision, but do not contain distance information in other regions. One useful way to reduce the redundancy in contact prediction evaluation is to select only one contact per residue (e.g. the evaluation results in Supplementary Table S3). However, since some residue may be involved in contacts in multiple contact clusters, this criterion can exclude important contacts as well. One complimentary approach to remedy the problem is to evaluate the coverage of prediction of contact clusters (Gao *et al.*, 2019).

Moreover, in the future, we will design more advanced deep learning architectures to predict more detailed inter-residue distance relationships (e.g. the distance distribution in finer distance intervals and even the real-number distance) and apply them to build protein structures from scratch, and rank protein structural models (Hou *et al.*, 2019).

## Supplementary Material

btz679_Supplementary_Data
