# Supplementary material for: Analysis of several key factors influencing deep learning-based inter-residue contact prediction
Source: Bioinformatics. 2019 Aug 30;36(4):1091–8. doi: 10.1093/bioinformatics/btz679 (PMC7703788; doi:10.1093/bioinformatics/btz679)
Supplement: btz679_Supplementary_Data [file btz679_supplementary_data.zip › supplementary_document.docx]

**Table S1.**The precision of top L/5 long-range contacts predicted by three coevolution-based methods using the alignments of three MULTICOM predictors

| **Target** | **Classification** | **CCMpred (%)** | | | **Freecontact (%)** | | | **PSICOV (%)** | | |
| --- | --- | --- | --- | --- | --- | --- | --- | --- | --- | --- |
|  |  | **NOVEL** | **CONSTRUCT** | **CLUSTER** | **NOVEL** | **CONSTRUCT** | **CLUSTER** | **NOVEL** | **CONSTRUCT** | **CLUSTER** |
| T0949-D1 | FM/TBM | 92.3 | 96.2 | 96.2 | 92.3 | 88.5 | 84.6 | 84.6 | 88.5 | 69.2 |
| T0950-D1 | FM | 1.5 | 1.5 | 1.5 | 1.5 | 1.5 | 1.5 | 1.5 | 1.5 | 2.9 |
| T0953s2-D1 | FM/TBM | 44.4 | 44.4 | 55.6 | 44.4 | 44.4 | 44.4 | 33.3 | 33.3 | 33.3 |
| T0953s2-D2 | FM | 68.0 | 68.0 | 64.0 | 48.0 | 48.0 | 52.0 | 48.0 | 48.0 | 48.0 |
| T0953s2-D3 | FM | 40.0 | 40.0 | 40.0 | 33.3 | 33.3 | 33.3 | 53.3 | 46.2 | 53.3 |
| T0957s1-D1 | FM | 22.7 | 22.7 | 22.7 | 4.6 | 4.6 | 9.1 | 18.2 | 18.2 | 18.2 |
| T0957s2-D1 | FM | 6.5 | 6.5 | 6.5 | 12.9 | 12.9 | 19.4 | 6.5 | 6.5 | 12.9 |
| T0958-D1 | FM/TBM | 26.7 | 26.7 | 26.7 | 13.3 | 13.3 | 0.0 | 13.3 | 13.3 | 20.0 |
| T0960-D2 | FM | 0.0 | 0.0 | 0 | 5.9 | 5.9 | 5.9 | 0.0 | 5.9 | 0.0 |
| T0963-D2 | FM | 6.3 | 6.3 | 6.3 | 12.5 | 12.5 | 12.5 | 6.3 | 6.3 | 6.3 |
| T0968s1-D1 | FM | 16.7 | 20.8 | 20.8 | 8.3 | 16.7 | 8.3 | 8.3 | 16.7 | 20.8 |
| T0968s2-D1 | FM | 17.4 | 17.4 | 13 | 26.1 | 17.4 | 13.0 | 8.7 | 17.4 | 8.7 |
| T0969-D1 | FM | 67.6 | 66.2 | 67.6 | 54.9 | 54.9 | 50.7 | 43.7 | 43.7 | 52.1 |
| T0970-D1 | FM/TBM | 11.8 | 17.7 | 5.9 | 5.9 | 11.8 | 5.9 | 17.7 | 5.9 | 11.8 |
| T0975-D1 | FM | 39.3 | 41.1 | 39.3 | 35.7 | 35.7 | 35.7 | 44.6 | 44.6 | 42.9 |
| T0978-D1 | FM/TBM | 39.8 | 53.0 | 61.5 | 49.4 | 61.5 | 60.2 | 43.4 | 56.6 | 60.2 |
| T0980s1-D1 | FM | 9.5 | 14.3 | 9.5 | 4.8 | 14.3 | 4.8 | 19.1 | 14.3 | 9.5 |
| T0981-D2 | FM | 0 | 0 | 0 | 0 | 0 | 0 | 0 | 0 | 0 |
| T0981-D3 | FM/TBM | 90.2 | 90.2 | 90.2 | 85.4 | 85.4 | 85.4 | 92.7 | 92.7 | 92.7 |
| T0986s1-D1 | FM/TBM | 38.9 | 33.3 | 27.8 | 44.4 | 27.8 | 38.9 | 22.2 | 27.8 | 22.2 |
| T0986s2-D1 | FM | 3.2 | 6.5 | 3.2 | 3.2 | 3.2 | 3.2 | 3.2 | 3.2 | 0.0 |
| T0987-D1 | FM | 10.8 | 10.8 | 13.5 | 2.7 | 2.7 | 0.0 | 13.5 | 13.5 | 13.5 |
| T0987-D2 | FM | 20.5 | 23.1 | 12.8 | 2.6 | 2.6 | 12.8 | 15.4 | 15.4 | 7.7 |
| T0989-D1 | FM | 48.2 | 51.9 | 51.9 | 40.7 | 40.7 | 44.4 | 40.7 | 33.3 | 40.7 |
| T0989-D2 | FM | 9.1 | 13.6 | 9.1 | 13.6 | 13.6 | 4.6 | 13.6 | 13.6 | 18.2 |
| T0990-D1 | FM | 6.7 | 6.7 | 6.7 | 6.7 | 6.7 | 0.0 | 6.7 | 6.7 | 6.7 |
| T0990-D2 | FM | 13.0 | 10.9 | 10.9 | 4.4 | 4.4 | 4.4 | 8.7 | 8.7 | 6.5 |
| T0990-D3 | FM | 4.7 | 7.0 | 4.7 | 2.3 | 2.3 | 0.0 | 4.7 | 4.7 | 4.7 |
| T0991-D1 | FM | 0 | 0 | 0 | 0 | 0 | 0 | 0 | 0 | 0 |
| T0992-D1 | FM/TBM | 57.1 | 61.9 | 76.2 | 42.9 | 57.1 | 66.7 | 61.9 | 42.9 | 66.7 |
| T0997-D1 | FM/TBM | 78.4 | 75.7 | 81.1 | 59.5 | 59.5 | 62.2 | 78.4 | 78.4 | 67.6 |
| T0998-D1 | FM | 9.1 | 9.1 | 9.1 | 9.1 | 9.1 | 9.1 | 9.1 | 0.0 | 9.1 |
| T1000-D2 | FM | 75.7 | 77.0 | 75.7 | 81.1 | 78.4 | 79.7 | 55.4 | 77.0 | 75.7 |
| T1001-D1 | FM | 3.6 | 10.7 | 7.1 | 0.0 | 0.0 | 0.0 | 0.0 | 0.0 | 0.0 |
| T1005-D1 | FM/TBM | 52.3 | 52.3 | 52.3 | 55.4 | 55.4 | 55.4 | 52.3 | 52.3 | 50.8 |
| T1008-D1 | FM/TBM | 0.0 | 0.0 | 0 | 6.7 | 0.0 | 13.3 | 0.0 | 0.0 | 6.7 |
| T1010-D1 | FM | 4.8 | 4.8 | 4.8 | 0.0 | 2.4 | 0.0 | 4.8 | 4.8 | 4.8 |
| T1015s1-D1 | FM | 55.6 | 44.4 | 61.1 | 38.9 | 38.9 | 38.9 | 22.2 | 27.8 | 44.4 |
| T1017s2-D1 | FM | 16.0 | 12.0 | 20 | 8.0 | 4.0 | 16.0 | 12.0 | 12.0 | 20.0 |
| T1019s1-D1 | FM/TBM | 16.7 | 8.3 | 16.7 | 16.7 | 16.7 | 25.0 | 8.3 | 16.7 | 25.0 |
| T1021s3-D1 | FM | 45.5 | 48.5 | 48.5 | 45.5 | 48.5 | 48.5 | 39.4 | 42.4 | 39.4 |
| T1021s3-D2 | FM | 10.5 | 0.0 | 0 | 10.5 | 0.0 | 0.0 | 21.1 | 10.5 | 10.5 |
| T1022s1-D1 | FM | 71.0 | 77.4 | 74.2 | 64.5 | 54.8 | 58.1 | 71.0 | 48.4 | 74.2 |
| Average |  | 29.1 | 29.7 | 30.1 | 25.5 | 25.4 | 25.8 | 25.8 | 25.6 | 27.4 |

**Table S2.** Neff of the alignments of three methods

| **Target** | **L** | **Classification** | **MULTICOM-NOVEL_Neff** | **MULTICOM-CONSTRUCT_Neff** | **MULTICOM-CLUSTER_Neff** |
| --- | --- | --- | --- | --- | --- |
| T0949-D1 | 139 | FM/TBM | 909.3 | 1154.8 | 524.2 |
| T0950-D1 | 342 | FM | 4336.7 | 4248.5 | 4261.6 |
| T0953s2-D1 | 44 | FM/TBM | 30.8 | 27.4 | 27.8 |
| T0957s2-D1 | 155 | FM | 45.3 | 44.4 | 31.8 |
| T0958-D1 | 77 | FM/TBM | 16.5 | 16.5 | 18.6 |
| T0960-D2 | 84 | FM | 218.9 | 209.2 | 216.3 |
| T0963-D2 | 82 | FM | 212.4 | 212.4 | 519.9 |
| T0968s1-D1 | 119 | FM | 73.5 | 86.7 | 83.9 |
| T0968s2-D1 | 116 | FM | 191.6 | 217.9 | 212.4 |
| T0969-D1 | 354 | FM | 279.1 | 279.1 | 340.3 |
| T0970-D1 | 97 | FM/TBM | 17.9 | 35.6 | 24.0 |
| T0975-D1 | 293 | FM | 8239.6 | 7974.8 | 7998.7 |
| T0978-D1 | 413 | FM/TBM | 190.3 | 375.3 | 276.9 |
| T0980s1-D1 | 105 | FM | 46.4 | 46.4 | 45.4 |
| T0981-D3 | 203 | FM/TBM | 2486.6 | 2486.6 | 2497.8 |
| T0986s1-D1 | 92 | FM/TBM | 109.9 | 155.8 | 144.3 |
| T0986s2-D1 | 155 | FM | 94.9 | 85.1 | 90.5 |
| T0987-D1 | 185 | FM | 26.3 | 26.3 | 26.5 |
| T0987-D2 | 207 | FM | 26.6 | 26.5 | 27.3 |
| T0989-D1 | 134 | FM | 232.2 | 224.4 | 230.3 |
| T0989-D2 | 112 | FM | 84.8 | 84.8 | 84.4 |
| T0990-D1 | 76 | FM | 30.3 | 30.0 | 30.2 |
| T0990-D3 | 213 | FM | 36.5 | 36.2 | 36.2 |
| T0992-D1 | 107 | FM/TBM | 452.9 | 523.1 | 585.6 |
| T0997-D1 | 185 | FM/TBM | 365.0 | 365.0 | 368.1 |
| T0998-D1 | 166 | FM | 7048.3 | 7048.3 | 7216.1 |
| T1000-D2 | 431 | FM | 336.5 | 371.5 | 384.2 |
| T1001-D1 | 139 | FM | 8.2 | 8.2 | 16.2 |
| T1005-D1 | 326 | FM/TBM | 1348.6 | 1302.2 | 1328.1 |
| T1008-D1 | 77 | FM/TBM | 10.0 | 43.1 | 5.0 |
| T1010-D1 | 210 | FM | 230.5 | 213.8 | 222.3 |
| T1015s1-D1 | 88 | FM | 146.6 | 167.9 | 165.9 |
| T1017s2-D1 | 128 | FM | 58.0 | 63.2 | 63.8 |
| T1019s1-D1 | 58 | FM/TBM | 24.7 | 348.0 | 414.7 |
| T1021s3-D1 | 178 | FM | 625.7 | 925.0 | 927.2 |
| T1021s3-D2 | 101 | FM | 47.9 | 124.7 | 125.7 |
| T1022s1-D1 | 156 | FM | 536.2 | 760.3 | 684.5 |
| **Average** |  |  | 788.5 | 820.2 | 817.7 |

**Table S3.** One residue evaluation on 108 domains of CASP13 targets

| **Method** | **Short-range (%)** | | | | | | **Medium-range (%)** | | | | | **Long-range (%)** | | | | |
| --- | --- | --- | --- | --- | --- | --- | --- | --- | --- | --- | --- | --- | --- | --- | --- | --- |
|  | **Top-L/5** | | **Top-L/2** | | **Top-L** | **Top-L/5** | | **Top-L/2** | **Top-L** | **Top-L/5** | | | | **Top-L/2** | **Top-L** | |
| **MULTICOM-CLUSTER** | 41.1 | 25.1 | | 15.1 | | 38.9 | | 25.4 | 16.5 | | 37.9 | | 25.7 | | | 18.3 |
| **MULTICOM-CONSTRUCT** | 41.3 | 25.0 | | 15.0 | | 37.9 | | 25.1 | 16.3 | | 36.6 | | 25.6 | | | 18.0 |
| **MULTICOM-NOVEL** | 39.0 | 24.1 | | 14.7 | | 36.1 | | 23.7 | 15.4 | | 33.6 | | 23.7 | | | 16.7 |

**Table S4.** Contact prediction precision on 43 CASP13 FM and FM-TBM domains

| **Method** | **Short-range (%)** | | | | | | **Medium-range (%)** | | | | | **Long-range (%)** | | | | |
| --- | --- | --- | --- | --- | --- | --- | --- | --- | --- | --- | --- | --- | --- | --- | --- | --- |
|  | **Top-L/5** | | **Top-L/2** | | **Top-L** | **Top-L/5** | | **Top-L/2** | **Top-L** | **Top-L/5** | | | | **Top-L/2** | **Top-L** | |
| **RaptorX [1]** | 67.5 | 48.9 | | 31.0 | | 72.9 | | 54.6 | 37.9 | | 74.8 | | 61.3 | | | 48.2 |
| **Restriplet [2]** | 69.1 | 47.4 | | 30.4 | | 74.7 | | 55.1 | 37.4 | | 68.7 | | 58.4 | | | 45.3 |
| **DMP [3]** | 64.5 | 45.3 | | 30.3 | | 69.3 | | 53.0 | 36.4 | | 66.5 | | 53.5 | | | 42.0 |
| **MULTICOM-CLUSTER** | 52.4 | 38.4 | | 26.7 | | 55.8 | | 42.0 | 29.6 | | 51.0 | | 40.9 | | | 31.6 |
| **MULTICOM-CONSTRUCT** | 53.1 | 37.9 | | 26.0 | | 53.7 | | 41.4 | 28.4 | | 47.9 | | 39.2 | | | 30.2 |
| **MULTICOM-NOVEL** | 51.8 | 37.3 | | 25.1 | | 51.8 | | 38.3 | 27.1 | | 49.7 | | 38.7 | | | 30.1 |
| **CCMpred** | 21.7 | 15.4 | | 12.0 | | 23.1 | | 15.4 | 12.3 | | 29.1 | | 21.9 | | | 15.7 |
| **Freecontact** | 19.1 | 14.4 | | 11.1 | | 20.6 | | 15.5 | 11.6 | | 25.5 | | 18.1 | | | 13.3 |
| **PSICOV** | 19.0 | 13.7 | | 12.0 | | 19.3 | | 13.6 | 12.0 | | 23.6 | | 17.4 | | | 12.5 |

**Table S5.** Contact prediction precision and runtime on 345 CulledPDB targets

| **CulledPDB** | **MULTICOM-NOVEL** | **MULTICOM-CONSTRUCT** | **MULTICOM-CLUSTER** |
| --- | --- | --- | --- |
| **Top-L/5 (%)** | 72.9 | 76.3 | 76.3 |
| **Top-L/2 (%)** | 62.8 | 65.7 | 65.5 |
| **Top-L (%)** | 50.3 | 51.8 | 51.8 |
| **Runtime(secs)** | 6534 | 6959 | 7152 |

**Table S6.** Change of precision of the long-range top L/5 contact predictions MULTICOM-CLUSTER with ab initio domain parsing and template-based domain parsing for CASP13 FM, FM/TBM and TBM-hard targets with respect to the precision of not using domain parsing. 0: no change; >0: increase of percentage points of precision; <0: decrease of percentage of points of precision

| Domain | Classification | Ab initio domain parsing | Template-based domain parsing |
| --- | --- | --- | --- |
| T0949-D1 | FM/TBM | 0 | 0 |
| T0950-D1 | FM | 4.41 | 0 |
| T0953s2-D1 | FM/TBM | 0 | 0 |
| T0953s2-D2 | FM | 0 | 0 |
| T0953s2-D3 | FM | 0 | 0 |
| T0955-D1 | FM/TBM | 0 | 0 |
| T0957s1-D1 | FM | 0 | 0 |
| T0957s2-D1 | FM | 0 | 0 |
| T0958-D1 | FM/TBM | 0 | 0 |
| T0960-D2 | FM | 0 | 0 |
| T0963-D2 | FM | 0 | 0 |
| T0968s1-D1 | FM | 0 | 0 |
| T0968s2-D1 | FM | 0 | 0 |
| T0969-D1 | FM | -4.23 | -4.23 |
| T0970-D1 | FM/TBM | 0 | 0 |
| T0975-D1 | FM | 0 | 0 |
| T0978-D1 | FM/TBM | 0 | 0 |
| T0980s1-D1 | FM | 0 | 0 |
| T0981-D2 | FM | 0 | -18.75 |
| T0981-D3 | FM/TBM | 0 | 0 |
| T0986s1-D1 | FM/TBM | 0 | 0 |
| T0986s2-D1 | FM | 0 | 0 |
| T0987-D1 | FM | 0 | 0 |
| T0987-D2 | FM | 0 | 0 |
| T0989-D1 | FM | 0 | 0 |
| T0989-D2 | FM | 13.64 | 40.91 |
| T0990-D1 | FM | 0 | 0 |
| T0990-D2 | FM | 4.35 | 0 |
| T0990-D3 | FM | -9.3 | 0 |
| T0991-D1 | FM | 0 | 0 |
| T0992-D1 | FM/TBM | 0 | 0 |
| T0997-D1 | FM/TBM | 0 | 0 |
| T0998-D1 | FM | 0 | 0 |
| T1000-D2 | FM | 0 | 0 |
| T1001-D1 | FM | 0 | 0 |
| T1005-D1 | FM/TBM | 1.54 | 0 |
| T1008-D1 | FM/TBM | 0 | 0 |
| T1010-D1 | FM | 0 | 2.38 |
| T1015s1-D1 | FM | 0 | 0 |
| T1017s2-D1 | FM | 0 | 0 |
| T1019s1-D1 | FM/TBM | 0 | 0 |
| T1021s3-D1 | FM | 0 | 0 |
| T1021s3-D2 | FM | -10.52 | 5.27 |
| T1022s1-D1 | FM | 0 | -9.67 |
| T0954-D1 | TBM-hard | 0 | 0 |
| T0957s1-D2 | TBM-hard | 0 | 0 |
| T0959-D1 | TBM-hard | 0 | 0 |
| T0960-D3 | TBM-hard | 0 | 55.56 |
| T0963-D3 | TBM-hard | 0 | 0 |
| T0964-D1 | TBM-hard | 57.89 | 89.47 |
| T0965-D1 | TBM-hard | 0 | 0 |
| T0966-D1 | TBM-hard | 0 | 0 |
| T0981-D1 | TBM-hard | 29.41 | 35.29 |
| T0981-D4 | TBM-hard | 36.37 | 40.91 |
| T0981-D5 | TBM-hard | 44 | 92 |
| T0982-D2 | TBM-hard | 0 | 0 |
| T0985-D1 | TBM-hard | -9.53 | 0 |
| T0999-D2 | TBM-hard | 0 | 57.15 |
| T1009-D1 | TBM-hard | 0 | 0 |
| T1011-D1 | TBM-hard | 0 | 0 |
| T1015s2-D1 | TBM-hard | 0 | 0 |
| T1021s1-D1 | TBM-hard | 0 | 0 |
| T1021s2-D1 | TBM-hard | 0 | 0 |
| T1022s1-D2 | TBM-hard | 0 | 0 |
| T1022s2-D1 | TBM-hard | 0 | 0 |


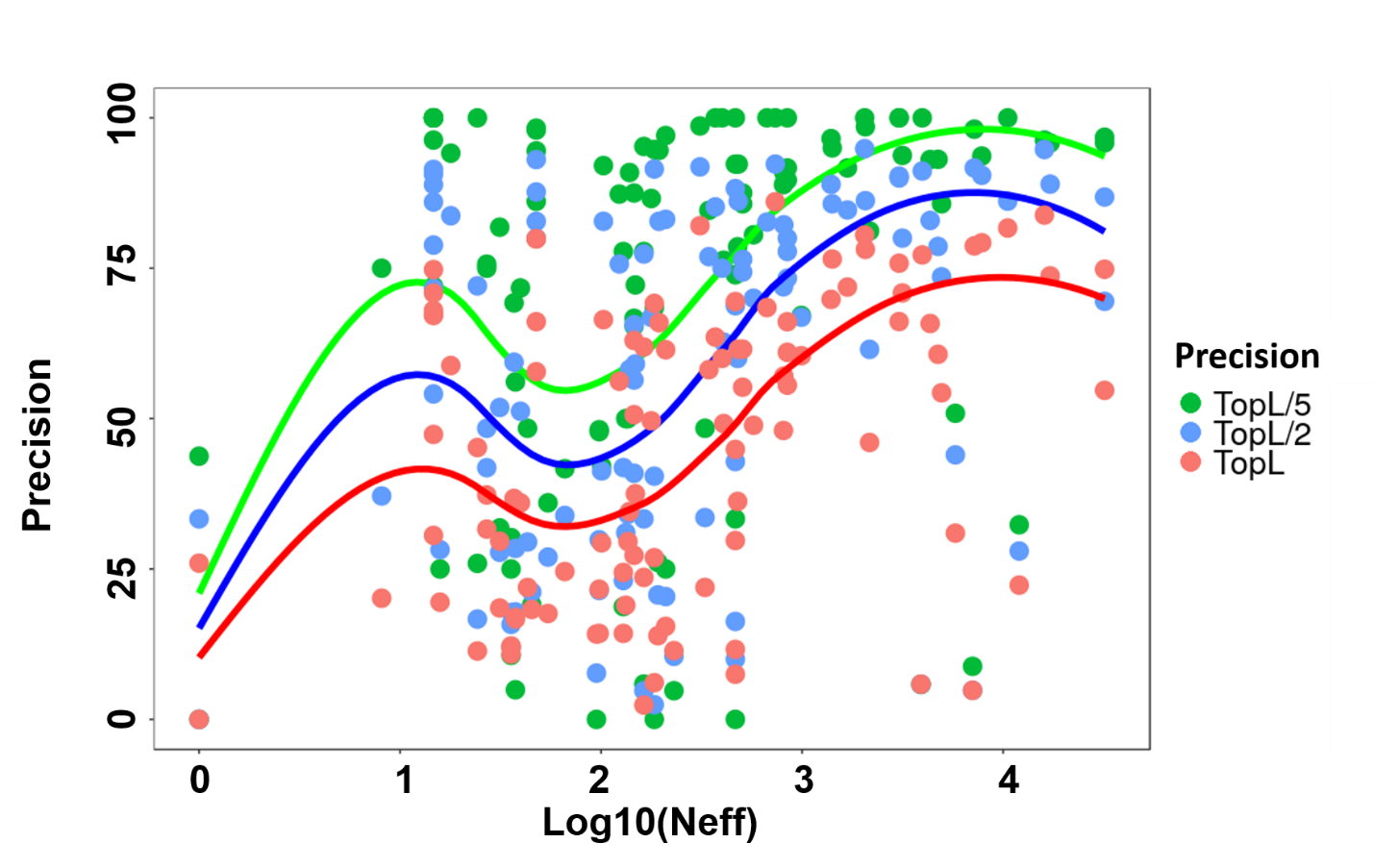


**Figure S1.** Plot of contact prediction precision against log10(Neff) of multiple sequence alignments
for 108 CASP13 domains for MULTICOM-NOVEL. Dots with three colors represent
different top ranked contacts. The curve is the LOESS line fitting the dots.

References:

1. Xu, J., & Wang, S. (2019). Analysis of distance-based protein structure prediction by deep learning in CASP13. bioRxiv, 624460
2. Li, Y., Hu, J., Zhang, C., Yu, D. J., & Zhang, Y. (2019). ResPRE: high-accuracy protein contact prediction by coupling precision matrix with deep residual neural networks. Bioinformatics
3. Kandathil, S. M., Greener, J. G., & Jones, D. T. (2019). Prediction of inter-residue contacts with DeepMetaPSICOV in CASP13. BioRxiv, 586800
