# Supplementary material for: Analysis of several key factors influencing deep learning-based inter-residue contact prediction
Source: Bioinformatics. 2019 Aug 30;36(4):1091–8. doi: 10.1093/bioinformatics/btz679 (PMC7703788; doi:10.1093/bioinformatics/btz679)
Supplement: btz679_Supplementary_Data [file btz679_supplementary_data.zip › supplementary_revised_Cheng.docx]

**Table S1.**The precision of top L/5 long-range contacts predicted by three coevolution-based methods using the alignments of three MULTICOM predictors

| **Target** | **Classification** | **CCMpred (%)** | | | **Freecontact (%)** | | | **PSICOV (%)** | | |
| --- | --- | --- | --- | --- | --- | --- | --- | --- | --- | --- |
|  |  | **NOVEL** | **CONSTRUCT** | **CLUSTER** | **NOVEL** | **CONSTRUCT** | **CLUSTER** | **NOVEL** | **CONSTRUCT** | **CLUSTER** |
| T0949-D1 | FM/TBM | 92.3 | 96.2 | 96.2 | 92.3 | 88.5 | 84.6 | 84.6 | 88.5 | 69.2 |
| T0950-D1 | FM | 1.5 | 1.5 | 1.5 | 1.5 | 1.5 | 1.5 | 1.5 | 1.5 | 2.9 |
| T0953s2-D1 | FM/TBM | 44.4 | 44.4 | 55.6 | 44.4 | 44.4 | 44.4 | 33.3 | 33.3 | 33.3 |
| T0953s2-D2 | FM | 68.0 | 68.0 | 64.0 | 48.0 | 48.0 | 52.0 | 48.0 | 48.0 | 48.0 |
| T0953s2-D3 | FM | 40.0 | 40.0 | 40.0 | 33.3 | 33.3 | 33.3 | 53.3 | 46.2 | 53.3 |
| T0957s1-D1 | FM | 22.7 | 22.7 | 22.7 | 4.6 | 4.6 | 9.1 | 18.2 | 18.2 | 18.2 |
| T0957s2-D1 | FM | 6.5 | 6.5 | 6.5 | 12.9 | 12.9 | 19.4 | 6.5 | 6.5 | 12.9 |
| T0958-D1 | FM/TBM | 26.7 | 26.7 | 26.7 | 13.3 | 13.3 | 0.0 | 13.3 | 13.3 | 20.0 |
| T0960-D2 | FM | 0.0 | 0.0 | 0 | 5.9 | 5.9 | 5.9 | 0.0 | 5.9 | 0.0 |
| T0963-D2 | FM | 6.3 | 6.3 | 6.3 | 12.5 | 12.5 | 12.5 | 6.3 | 6.3 | 6.3 |
| T0968s1-D1 | FM | 16.7 | 20.8 | 20.8 | 8.3 | 16.7 | 8.3 | 8.3 | 16.7 | 20.8 |
| T0968s2-D1 | FM | 17.4 | 17.4 | 13 | 26.1 | 17.4 | 13.0 | 8.7 | 17.4 | 8.7 |
| T0969-D1 | FM | 67.6 | 66.2 | 67.6 | 54.9 | 54.9 | 50.7 | 43.7 | 43.7 | 52.1 |
| T0970-D1 | FM/TBM | 11.8 | 17.7 | 5.9 | 5.9 | 11.8 | 5.9 | 17.7 | 5.9 | 11.8 |
| T0975-D1 | FM | 39.3 | 41.1 | 39.3 | 35.7 | 35.7 | 35.7 | 44.6 | 44.6 | 42.9 |
| T0978-D1 | FM/TBM | 39.8 | 53.0 | 61.5 | 49.4 | 61.5 | 60.2 | 43.4 | 56.6 | 60.2 |
| T0980s1-D1 | FM | 9.5 | 14.3 | 9.5 | 4.8 | 14.3 | 4.8 | 19.1 | 14.3 | 9.5 |
| T0981-D2 | FM | 0 | 0 | 0 | 0 | 0 | 0 | 0 | 0 | 0 |
| T0981-D3 | FM/TBM | 90.2 | 90.2 | 90.2 | 85.4 | 85.4 | 85.4 | 92.7 | 92.7 | 92.7 |
| T0986s1-D1 | FM/TBM | 38.9 | 33.3 | 27.8 | 44.4 | 27.8 | 38.9 | 22.2 | 27.8 | 22.2 |
| T0986s2-D1 | FM | 3.2 | 6.5 | 3.2 | 3.2 | 3.2 | 3.2 | 3.2 | 3.2 | 0.0 |
| T0987-D1 | FM | 10.8 | 10.8 | 13.5 | 2.7 | 2.7 | 0.0 | 13.5 | 13.5 | 13.5 |
| T0987-D2 | FM | 20.5 | 23.1 | 12.8 | 2.6 | 2.6 | 12.8 | 15.4 | 15.4 | 7.7 |
| T0989-D1 | FM | 48.2 | 51.9 | 51.9 | 40.7 | 40.7 | 44.4 | 40.7 | 33.3 | 40.7 |
| T0989-D2 | FM | 9.1 | 13.6 | 9.1 | 13.6 | 13.6 | 4.6 | 13.6 | 13.6 | 18.2 |
| T0990-D1 | FM | 6.7 | 6.7 | 6.7 | 6.7 | 6.7 | 0.0 | 6.7 | 6.7 | 6.7 |
| T0990-D2 | FM | 13.0 | 10.9 | 10.9 | 4.4 | 4.4 | 4.4 | 8.7 | 8.7 | 6.5 |
| T0990-D3 | FM | 4.7 | 7.0 | 4.7 | 2.3 | 2.3 | 0.0 | 4.7 | 4.7 | 4.7 |
| T0991-D1 | FM | 0 | 0 | 0 | 0 | 0 | 0 | 0 | 0 | 0 |
| T0992-D1 | FM/TBM | 57.1 | 61.9 | 76.2 | 42.9 | 57.1 | 66.7 | 61.9 | 42.9 | 66.7 |
| T0997-D1 | FM/TBM | 78.4 | 75.7 | 81.1 | 59.5 | 59.5 | 62.2 | 78.4 | 78.4 | 67.6 |
| T0998-D1 | FM | 9.1 | 9.1 | 9.1 | 9.1 | 9.1 | 9.1 | 9.1 | 0.0 | 9.1 |
| T1000-D2 | FM | 75.7 | 77.0 | 75.7 | 81.1 | 78.4 | 79.7 | 55.4 | 77.0 | 75.7 |
| T1001-D1 | FM | 3.6 | 10.7 | 7.1 | 0.0 | 0.0 | 0.0 | 0.0 | 0.0 | 0.0 |
| T1005-D1 | FM/TBM | 52.3 | 52.3 | 52.3 | 55.4 | 55.4 | 55.4 | 52.3 | 52.3 | 50.8 |
| T1008-D1 | FM/TBM | 0.0 | 0.0 | 0 | 6.7 | 0.0 | 13.3 | 0.0 | 0.0 | 6.7 |
| T1010-D1 | FM | 4.8 | 4.8 | 4.8 | 0.0 | 2.4 | 0.0 | 4.8 | 4.8 | 4.8 |
| T1015s1-D1 | FM | 55.6 | 44.4 | 61.1 | 38.9 | 38.9 | 38.9 | 22.2 | 27.8 | 44.4 |
| T1017s2-D1 | FM | 16.0 | 12.0 | 20 | 8.0 | 4.0 | 16.0 | 12.0 | 12.0 | 20.0 |
| T1019s1-D1 | FM/TBM | 16.7 | 8.3 | 16.7 | 16.7 | 16.7 | 25.0 | 8.3 | 16.7 | 25.0 |
| T1021s3-D1 | FM | 45.5 | 48.5 | 48.5 | 45.5 | 48.5 | 48.5 | 39.4 | 42.4 | 39.4 |
| T1021s3-D2 | FM | 10.5 | 0.0 | 0 | 10.5 | 0.0 | 0.0 | 21.1 | 10.5 | 10.5 |
| T1022s1-D1 | FM | 71.0 | 77.4 | 74.2 | 64.5 | 54.8 | 58.1 | 71.0 | 48.4 | 74.2 |
| Average |  | 29.1 | 29.7 | 30.1 | 25.5 | 25.4 | 25.8 | 25.8 | 25.6 | 27.4 |

**Table S2.** Neff of the alignments of three methods

| **Target** | **L** | **Classification** | **MULTICOM-NOVEL_Neff** | **MULTICOM-CONSTRUCT_Neff** | **MULTICOM-CLUSTER_Neff** |
| --- | --- | --- | --- | --- | --- |
| T0949-D1 | 139 | FM/TBM | 909.3 | 1154.8 | 524.2 |
| T0950-D1 | 342 | FM | 4336.7 | 4248.5 | 4261.6 |
| T0953s2-D1 | 44 | FM/TBM | 30.8 | 27.4 | 27.8 |
| T0957s2-D1 | 155 | FM | 45.3 | 44.4 | 31.8 |
| T0958-D1 | 77 | FM/TBM | 16.5 | 16.5 | 18.6 |
| T0960-D2 | 84 | FM | 218.9 | 209.2 | 216.3 |
| T0963-D2 | 82 | FM | 212.4 | 212.4 | 519.9 |
| T0968s1-D1 | 119 | FM | 73.5 | 86.7 | 83.9 |
| T0968s2-D1 | 116 | FM | 191.6 | 217.9 | 212.4 |
| T0969-D1 | 354 | FM | 279.1 | 279.1 | 340.3 |
| T0970-D1 | 97 | FM/TBM | 17.9 | 35.6 | 24.0 |
| T0975-D1 | 293 | FM | 8239.6 | 7974.8 | 7998.7 |
| T0978-D1 | 413 | FM/TBM | 190.3 | 375.3 | 276.9 |
| T0980s1-D1 | 105 | FM | 46.4 | 46.4 | 45.4 |
| T0981-D3 | 203 | FM/TBM | 2486.6 | 2486.6 | 2497.8 |
| T0986s1-D1 | 92 | FM/TBM | 109.9 | 155.8 | 144.3 |
| T0986s2-D1 | 155 | FM | 94.9 | 85.1 | 90.5 |
| T0987-D1 | 185 | FM | 26.3 | 26.3 | 26.5 |
| T0987-D2 | 207 | FM | 26.6 | 26.5 | 27.3 |
| T0989-D1 | 134 | FM | 232.2 | 224.4 | 230.3 |
| T0989-D2 | 112 | FM | 84.8 | 84.8 | 84.4 |
| T0990-D1 | 76 | FM | 30.3 | 30.0 | 30.2 |
| T0990-D3 | 213 | FM | 36.5 | 36.2 | 36.2 |
| T0992-D1 | 107 | FM/TBM | 452.9 | 523.1 | 585.6 |
| T0997-D1 | 185 | FM/TBM | 365.0 | 365.0 | 368.1 |
| T0998-D1 | 166 | FM | 7048.3 | 7048.3 | 7216.1 |
| T1000-D2 | 431 | FM | 336.5 | 371.5 | 384.2 |
| T1001-D1 | 139 | FM | 8.2 | 8.2 | 16.2 |
| T1005-D1 | 326 | FM/TBM | 1348.6 | 1302.2 | 1328.1 |
| T1008-D1 | 77 | FM/TBM | 10.0 | 43.1 | 5.0 |
| T1010-D1 | 210 | FM | 230.5 | 213.8 | 222.3 |
| T1015s1-D1 | 88 | FM | 146.6 | 167.9 | 165.9 |
| T1017s2-D1 | 128 | FM | 58.0 | 63.2 | 63.8 |
| T1019s1-D1 | 58 | FM/TBM | 24.7 | 348.0 | 414.7 |
| T1021s3-D1 | 178 | FM | 625.7 | 925.0 | 927.2 |
| T1021s3-D2 | 101 | FM | 47.9 | 124.7 | 125.7 |
| T1022s1-D1 | 156 | FM | 536.2 | 760.3 | 684.5 |
| **Average** |  |  | 788.5 | 820.2 | 817.7 |

**Table S3.** One residue evaluation on 108 on 108 CASP13 targets

| **Method** | **Short-range (%)** | | | | | | **Medium-range (%)** | | | | | **Long-range (%)** | | | | |
| --- | --- | --- | --- | --- | --- | --- | --- | --- | --- | --- | --- | --- | --- | --- | --- | --- |
|  | **Top-L/5** | | **Top-L/2** | | **Top-L** | **Top-L/5** | | **Top-L/2** | **Top-L** | **Top-L/5** | | | | **Top-L/2** | **Top-L** | |
| **MULTICOM-CLUSTER** | 41.1 | 25.1 | | 15.1 | | 38.9 | | 25.4 | 16.5 | | 37.9 | | 25.7 | | | 18.3 |
| **MULTICOM-CONSTRUCT** | 41.3 | 25.0 | | 15.0 | | 37.9 | | 25.1 | 16.3 | | 36.6 | | 25.6 | | | 18.0 |
| **MULTICOM-NOVEL** | 39.0 | 24.1 | | 14.7 | | 36.1 | | 23.7 | 15.4 | | 33.6 | | 23.7 | | | 16.7 |

**Table S4.** Contact prediction precision on 43 CASP13 FM and FM-TBM domains

| **Method** | **Short-range (%)** | | | | | | **Medium-range (%)** | | | | | **Long-range (%)** | | | | |
| --- | --- | --- | --- | --- | --- | --- | --- | --- | --- | --- | --- | --- | --- | --- | --- | --- |
|  | **Top-L/5** | | **Top-L/2** | | **Top-L** | **Top-L/5** | | **Top-L/2** | **Top-L** | **Top-L/5** | | | | **Top-L/2** | **Top-L** | |
| **RaptorX [1]** | 67.5 | 48.9 | | 31.0 | | 72.9 | | 54.6 | 37.9 | | 74.8 | | 61.3 | | | 48.2 |
| **Restriplet [2]** | 69.1 | 47.4 | | 30.4 | | 74.7 | | 55.1 | 37.4 | | 68.7 | | 58.4 | | | 45.3 |
| **DMP [3]** | 64.5 | 45.3 | | 30.3 | | 69.3 | | 53.0 | 36.4 | | 66.5 | | 53.5 | | | 42.0 |
| **MULTICOM-CLUSTER** | 52.4 | 38.4 | | 26.7 | | 55.8 | | 42.0 | 29.6 | | 51.0 | | 40.9 | | | 31.6 |
| **MULTICOM-CONSTRUCT** | 53.1 | 37.9 | | 26.0 | | 53.7 | | 41.4 | 28.4 | | 47.9 | | 39.2 | | | 30.2 |
| **MULTICOM-NOVEL** | 51.8 | 37.3 | | 25.1 | | 51.8 | | 38.3 | 27.1 | | 49.7 | | 38.7 | | | 30.1 |
| **CCMpred** | 21.7 | 15.4 | | 12.0 | | 23.1 | | 15.4 | 12.3 | | 29.1 | | 21.9 | | | 15.7 |
| **Freecontact** | 19.1 | 14.4 | | 11.1 | | 20.6 | | 15.5 | 11.6 | | 25.5 | | 18.1 | | | 13.3 |
| **PSICOV** | 19.0 | 13.7 | | 12.0 | | 19.3 | | 13.6 | 12.0 | | 23.6 | | 17.4 | | | 12.5 |

**Table S5.** Contact prediction precision and runtime on 345 CulledPDB targets

| **CulledPDB** | **MULTICOM-NOVEL** | **MULTICOM-CONSTRUCT** | **MULTICOM-CLUSTER** |
| --- | --- | --- | --- |
| **Top-L/5 (%)** | 72.9 | 76.3 | 76.3 |
| **Top-L/2 (%)** | 62.8 | 65.7 | 65.5 |
| **Top-L (%)** | 50.3 | 51.8 | 51.8 |
| **Runtime(secs)** | 6534 | 6959 | 7152 |

We tested the performance of our three MULTICOM contact prediction methods on 345 targets from cullpdb dataset (sequence identity <= 25% and resolution <= 2.5 Å), which are released after August, 2018 (e.g. after the deep learning predictors were trained). We calculated the runtime for each method in Table S5. From the results in the table, we could observe that the average runtime increased from 6534(secs) to 7152(secs) as the contact predictor added more steps in generating multiple sequence alignment and extra domain parsing method. The 9.5% added cost in the runtime is still acceptable in many situations and it is worth adding those extra steps for helping improve the performance of contact prediction when the accuracy is more important.

**Table S6.** Change of precision of the long-range top L/5 contact predictions MULTICOM-CLUSTER with ab initio domain parsing and template-based domain parsing for CASP13 FM, FM/TBM and TBM-hard targets with respect to the precision of not using domain parsing.

| Domain | Classification | Ab initio domain parsing | Template-based domain parsing |
| --- | --- | --- | --- |
| T0949-D1 | FM/TBM | 0 | 0 |
| T0950-D1 | FM | 4.41 | 0 |
| T0953s2-D1 | FM/TBM | 0 | 0 |
| T0953s2-D2 | FM | 0 | 0 |
| T0953s2-D3 | FM | 0 | 0 |
| T0955-D1 | FM/TBM | 0 | 0 |
| T0957s1-D1 | FM | 0 | 0 |
| T0957s2-D1 | FM | 0 | 0 |
| T0958-D1 | FM/TBM | 0 | 0 |
| T0960-D2 | FM | 0 | 0 |
| T0963-D2 | FM | 0 | 0 |
| T0968s1-D1 | FM | 0 | 0 |
| T0968s2-D1 | FM | 0 | 0 |
| T0969-D1 | FM | -4.23 | -4.23 |
| T0970-D1 | FM/TBM | 0 | 0 |
| T0975-D1 | FM | 0 | 0 |
| T0978-D1 | FM/TBM | 0 | 0 |
| T0980s1-D1 | FM | 0 | 0 |
| T0981-D2 | FM | 0 | -18.75 |
| T0981-D3 | FM/TBM | 0 | 0 |
| T0986s1-D1 | FM/TBM | 0 | 0 |
| T0986s2-D1 | FM | 0 | 0 |
| T0987-D1 | FM | 0 | 0 |
| T0987-D2 | FM | 0 | 0 |
| T0989-D1 | FM | 0 | 0 |
| T0989-D2 | FM | 13.64 | 40.91 |
| T0990-D1 | FM | 0 | 0 |
| T0990-D2 | FM | 4.35 | 0 |
| T0990-D3 | FM | -9.3 | 0 |
| T0991-D1 | FM | 0 | 0 |
| T0992-D1 | FM/TBM | 0 | 0 |
| T0997-D1 | FM/TBM | 0 | 0 |
| T0998-D1 | FM | 0 | 0 |
| T1000-D2 | FM | 0 | 0 |
| T1001-D1 | FM | 0 | 0 |
| T1005-D1 | FM/TBM | 1.54 | 0 |
| T1008-D1 | FM/TBM | 0 | 0 |
| T1010-D1 | FM | 0 | 2.38 |
| T1015s1-D1 | FM | 0 | 0 |
| T1017s2-D1 | FM | 0 | 0 |
| T1019s1-D1 | FM/TBM | 0 | 0 |
| T1021s3-D1 | FM | 0 | 0 |
| T1021s3-D2 | FM | -10.52 | 5.27 |
| T1022s1-D1 | FM | 0 | -9.67 |
| T0954-D1 | TBM-hard | 0 | 0 |
| T0957s1-D2 | TBM-hard | 0 | 0 |
| T0959-D1 | TBM-hard | 0 | 0 |
| T0960-D3 | TBM-hard | 0 | 55.56 |
| T0963-D3 | TBM-hard | 0 | 0 |
| T0964-D1 | TBM-hard | 57.89 | 89.47 |
| T0965-D1 | TBM-hard | 0 | 0 |
| T0966-D1 | TBM-hard | 0 | 0 |
| T0981-D1 | TBM-hard | 29.41 | 35.29 |
| T0981-D4 | TBM-hard | 36.37 | 40.91 |
| T0981-D5 | TBM-hard | 44 | 92 |
| T0982-D2 | TBM-hard | 0 | 0 |
| T0985-D1 | TBM-hard | -9.53 | 0 |
| T0999-D2 | TBM-hard | 0 | 57.15 |
| T1009-D1 | TBM-hard | 0 | 0 |
| T1011-D1 | TBM-hard | 0 | 0 |
| T1015s2-D1 | TBM-hard | 0 | 0 |
| T1021s1-D1 | TBM-hard | 0 | 0 |
| T1021s2-D1 | TBM-hard | 0 | 0 |
| T1022s1-D2 | TBM-hard | 0 | 0 |
| T1022s2-D1 | TBM-hard | 0 | 0 |


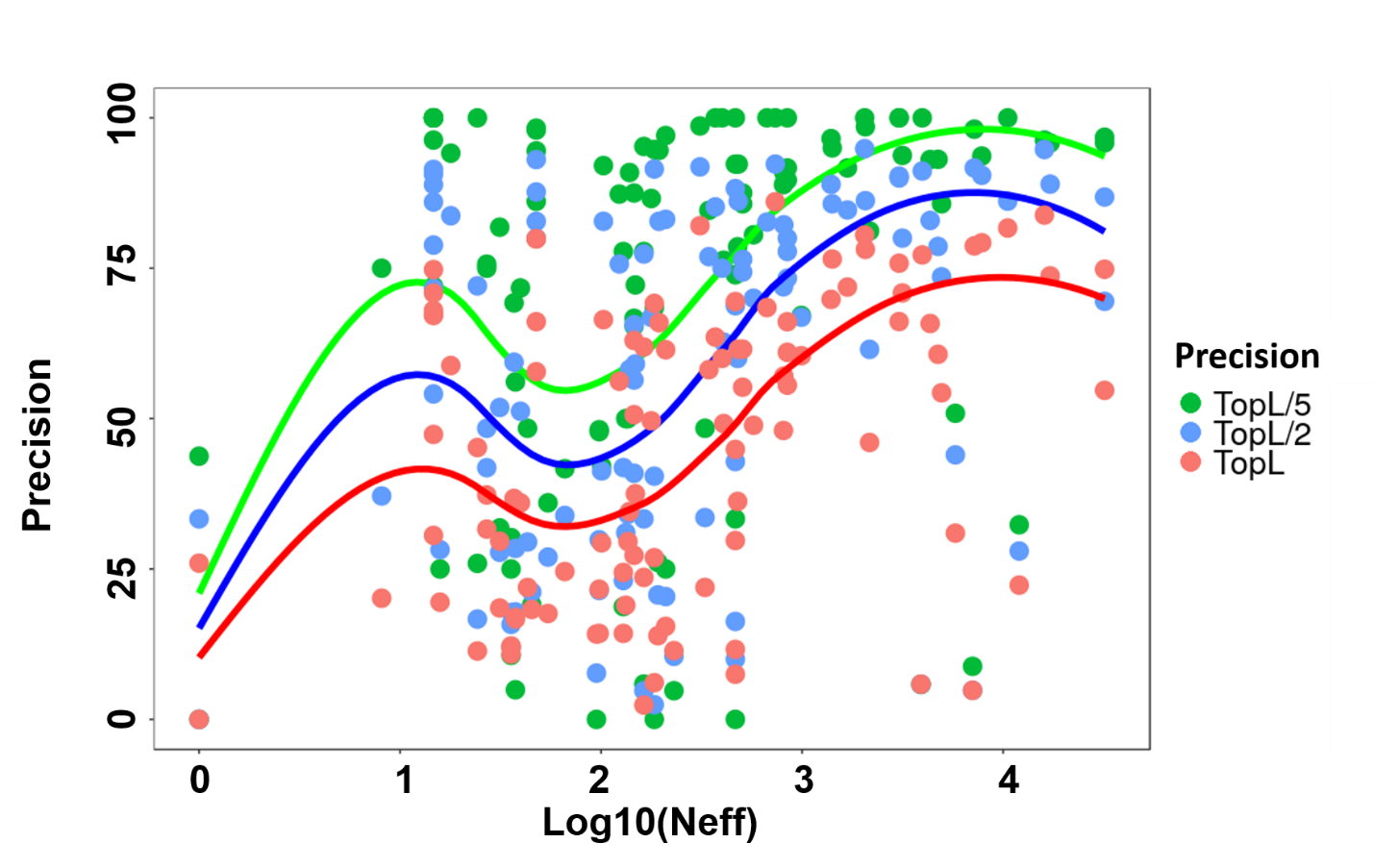


**Figure S1.** Plot of contact prediction precision against log10(Neff) of multiple sequence alignments
for 108 CASP13 domains for MULTICOM-NOVEL. Dots with three colors represent
different top ranked contacts. The curve is the LOESS line fitting the dots.

References:

1. Xu, J., & Wang, S. (2019). Analysis of distance-based protein structure prediction by deep learning in CASP13. bioRxiv, 624460
2. Li, Y., Hu, J., Zhang, C., Yu, D. J., & Zhang, Y. (2019). ResPRE: high-accuracy protein contact prediction by coupling precision matrix with deep residual neural networks. Bioinformatics
3. Kandathil, S. M., Greener, J. G., & Jones, D. T. (2019). Prediction of inter-residue contacts with DeepMetaPSICOV in CASP13. BioRxiv, 586800
